# P-760. Amikacin-Liposome Inhalation Suspension (ALIS) for Refractory MACLD: Simultaneous Analysis of Efficacy and Safety

**DOI:** 10.1093/ofid/ofae631.955

**Published:** 2025-01-29

**Authors:** Daniel Dorgan, Catherine Waweru, Kim Cocks, Rachael Lawrance, Matthias Hunger, Ankit Pahwa, Dayton Yuen, Monika Ciesielska, Leona Markson

**Affiliations:** University of Pennsylvania, Philadelphia, Pennsylvania; Insmed Incorporated, Clarksburg, Maryland; Adelphi Values, Bollington, England, United Kingdom; Adelphi Values, Bollington, England, United Kingdom; ICON Clinical Research, Dublin, Dublin, Ireland; ICON Clinical Research, Dublin, Dublin, Ireland; Insmed Incorporated, Clarksburg, Maryland; Insmed Incorporated, Clarksburg, Maryland; Insmed Incorporated, Clarksburg, Maryland

## Abstract

**Background:**

Patients with refractory Mycobacterium avium complex lung disease (rMACLD) represent a difficult to treat population, who have not achieved culture conversion (CC) despite prolonged treatment with multidrug antibacterial regimen (MDR). In the CONVERT trial, amikacin liposome inhalation suspension (ALIS) + MDR resulted in higher CC rates than MDR alone during the first 6 months of treatment (29% vs 9%) in patients that had positive cultures on MDR for at least 6 months. Adverse events (AEs) following treatment initiation were reported in 98% and 91% of patients in the ALIS+MDR and MDR alone arms, respectively, and were mostly of mild to moderate severity. A simultaneous assessment of efficacy and safety of treatment could be informative during clinical decision making. We adapted well-established Time Without Symptoms and Toxicity “TWiST” methodology to compare ALIS + MDR vs MDR alone, using a singular metric of time spent with CC and without severe AEs.

**Figure 1.**
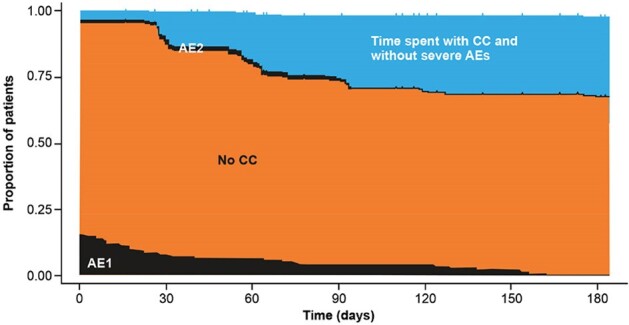
Partitioned survival curves for ALIS+MDR

**Methods:**

The time from randomization to month 6 in CONVERT was partitioned into 4 health states:

• Time spent with severe (≥ grade 3) AEs prior to CC (AE1)

• Time spent without CC, without severe AEs (No CC)

• Time spent with severe AEs after CC (AE2)

• Time spent with CC and without severe AEs (referred to as ideal health state)

Kaplan-Meier methods were used to estimate health state durations (days); p-values were estimated using bootstrapping with 10,000 replications.

**Figure 2.**
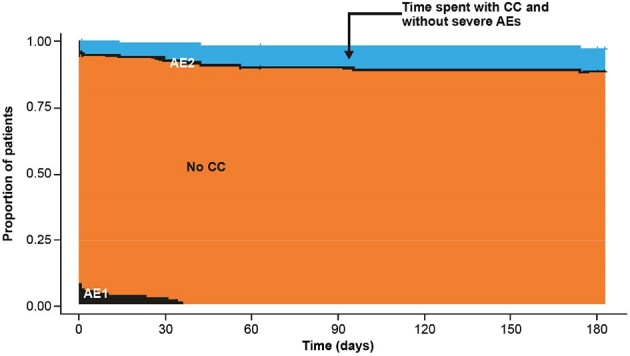
Partitioned survival curves for MDR alone

**Results:**

Mean follow-up was 180.2 and 179.7 days in the ALIS+MDR and MDR alone treatment arms, respectively. Patients in the ALIS+MDR arm spent a mean of 9.4 days with severe AEs prior to CC vs 1.4 with MDR alone (p < 0.001) (Figure 1, Figure 2). There was no statistically significant difference in the mean time spent with severe AEs following CC between treatment arms (ALIS+MDR vs MDR: 1.8 vs 0.6 days; p = 0.2). The mean number of days spent in the ideal health state was statistically significantly higher with ALIS+MDR vs MDR alone (38.8 vs 14.5 days; p < 0.001).

**Conclusion:**

Although there were more AEs on ALIS+MDR than MDR alone in the CONVERT trial, this simultaneous assessment of efficacy and safety demonstrates that ALIS+MDR was more likely to result in more time in the ideal health state of CC without severe AEs than MDR alone. These data may inform clinical decision-making in the treatment of rMACLD.

**Disclosures:**

**Daniel Dorgan, MD**, Insmed Incorporated: Advisor/Consultant **Catherine Waweru, PhD**, Insmed Incorporated: Employee|Insmed Incorporated: Stocks/Bonds (Public Company) **Kim Cocks, PhD**, Adelphi Values: Employee **Rachael Lawrance, BSc**, Adelphi Values: Employee **Matthias Hunger, MSc**, ICON Clinical Research: Employee **Ankit Pahwa, MS**, ICON Clinical Research: Employee **Dayton Yuen, PharmD**, Insmed Incorporated: Employee|Insmed Incorporated: Stocks/Bonds (Public Company) **Monika Ciesielska, MA**, Insmed Incorporated: Employee|Insmed Incorporated: Stocks/Bonds (Public Company) **Leona Markson, ScD**, Insmed Incorporated: Employee|Insmed Incorporated: Stocks/Bonds (Public Company)

